# Risk Factors for Cervical Intraepithelial Neoplasia in HIV-Infected Women on Antiretroviral Treatment in Côte d'Ivoire, West Africa

**DOI:** 10.1371/journal.pone.0090625

**Published:** 2014-03-04

**Authors:** Antoine Jaquet, Apollinaire Horo, Didier K. Ekouevi, Badian Toure, Patrick A. Coffie, Benjamin Effi, Severin Lenaud, Eugene Messou, Albert Minga, Annie J. Sasco, François Dabis, the IeDEA West Africa Collaboration

**Affiliations:** 1 Université Bordeaux, ISPED, Centre INSERM U897-Epidémiologie-Biostatistique, Bordeaux, France; 2 INSERM, ISPED, Centre INSERM U897- Epidémiologie-Biostatistique, Bordeaux, France; 3 Service de Gynécologie Obstétrique, Centre Hospitalier Universitaire (CHU) de Yopougon, Abidjan, Côte d'Ivoire; 4 Programme PACCI, CHU de Treichville, Abidjan, Côte d'Ivoire; 5 Service d'Anatomo-Pathologie, CHU de Treichville, Abidjan, Côte d'Ivoire; 6 CePReF, ACONDA, Abidjan, Côte d'Ivoire; 7 Centre Médical de Suivi de Donneurs de Sang/CNTS/PRIMO-CI, Abidjan, Cote d'Ivoire; University of Pittsburgh Center for Vaccine Research, United States of America

## Abstract

**Background:**

Facing the dual burden of invasive cervical cancer and HIV in sub-Saharan Africa, the identification of preventable determinants of Cervical Intraepithelial Neoplasia (CIN) in HIV-infected women is of paramount importance.

**Methods:**

A cervical cancer screening based on visual inspection methods was proposed to HIV-infected women in care in Abidjan, Côte d'Ivoire. Positively screened women were referred for a colposcopy to a gynaecologist who performed directed biopsies.

**Results:**

Of the 2,998 HIV-infected women enrolled, 132 (4.4%) CIN of any grade (CIN+) were identified. Women had been followed-up for a median duration of three years [IQR: 1–5] and 76% were on antiretroviral treatment (ART). Their median most recent CD4 count was 452 [IQR: 301–621] cells/mm^3^. In multivariate analysis, CIN+ was associated with a most recent CD4 count >350 cells/mm^3^ (OR: 0.3; 95% CI: 0.2–0.6) or ≥200–350 cells/mm^3^ (OR 0.6; 95% CI 0.4–1.0) (Ref: <200 cells/mm^3^ CD4) (p<10^−4^).

**Conclusions:**

The presence of CIN+ is less common among HIV-infected women with limited or no immune deficiency. Despite the potential impact of immunological recovery on the reduction of premalignant cervical lesions through the use of ART, cervical cancer prevention, including screening and vaccination remains a priority in West Africa while ART is rolled-out.

## Introduction

Since 2002, the number of HIV-infected patients accessing to antiretroviral treatment (ART) has dramatically increased with approximately half of those eligible to ART already covered in sub-Saharan Africa in 2010 [1]. As the ART scale-up continues to improve the life expectancy of HIV-infected individuals [2], a focus on long-term case management is needed, especially in women who account for approximately two-thirds of those receiving ART in sub-Saharan Africa. According to a 2012 estimates, invasive cervical cancer (ICC) is a leading cause of cancer in women in sub-Saharan Africa with an annual incidence of 93,000 new cases and 57,000 deaths [3]. Virtually, all ICC are caused by an infection with a high-risk type of Human Papillomavirus (HPV) which is now recognised as its necessary cofactor [4]. Although a great proportion of the sexually active population acquires HPV infection during their life, only a limited proportion harbours persistent HPV infection that evolves toward ICC and the factors modulating this process are poorly understood. Previous reports from case-control and cohort studies have linked HIV infection with ICC and its precursors in sub-Saharan Africa in a time of limited access to ART [5]–[7]. However, there is limited information regarding factors influencing the occurrence of cervical premalignant and malignant lesions in the ART era. The purpose of the present study was to identify determinants of cervical intraepithelial neoplasia (CIN) among HIV-infected women in care and attending a cervical cancer screening program in Côte d'Ivoire, West Africa. We hypothesized that immune recovery mediated by ART use would reduce the CIN prevalence.

## Methods

### Study population

A cervical screening program based on visual inspection of the cervix was organized in HIV clinics participating to the International epidemiological Database to Evaluate AIDS (IeDEA) West Africa collaboration (http://mereva.net/iedea) in Abidjan, the economic capital of Côte d'Ivoire. The cervical cancer screening procedures as well as its main outcomes have been previously described elsewhere [8]. Briefly, from August 2009 to November 2010, an itinerant team composed of three trained midwives and a senior gynaecologist was in charge of sequentially proposing cervical screening to all HIV-infected women in three urban HIV clinics that volunteered to participate. Women who had no previous cervical cancer or total hysterectomy, aged [25–65] years and no pregnancy >20 weeks were eligible for enrolment. After obtaining their written consent, midwives collected with a structured questionnaire information related to socio-demographic characteristics (age, formal education, marital status, tobacco use) and reproductive behaviours (age at first intercourse, lifetime number of sexual partners, contraceptive use and parity). HIV follow-up (CD4 count at first consultation and most recent CD4 count at cervical screening consultation, ART initiated and duration) were abstracted from the medical charts. Information related to the HIV type (HIV-1, HIV-2 or dually reactive) was extracted from the IeDEA West Africa database. Visual inspection of the cervix with acetic acid (VIA) and with Lugol's iodine (VILI) was performed during a standard gynaecological examination. Results of VIA and VILI were both classified as negative, positive or presumptive of ICC according to the International Agency for Research on Cancer (IARC) training manual [9]. Positively screened women (VIA+ and/or VILI+) were scheduled for colposcopy, carried out by a trained gynaecologist who performed directed biopsies in case of positive findings with colposcopy. Histopathological findings were categorized into five classes: normal or non-neoplasic changes, CIN of grade 1 including HPV changes, CIN of grade 2, CIN of grade 3 and invasive cancer as described elsewhere [10]. A written and informed consent was obtained from all the study participants for their participation to the cervical cancer screening procedure. Prior to their inclusion, women were orally counseled about the risk of cervical cancer and the potential benefits of an early detection. The entire screening procedure was free of charge. A second written informed consent was obtained from all women identified through screening and addressed for a colposcopic examination. Women with CIN+ were offered appropriate follow-up and treatment according to local recommendations. The treatment in case of CIN+ was free of charge. This study was approved by the national ethic committee of the Ministry of Health in Côte d'Ivoire.

### Statistical analysis

Women characteristics were compared first according to the presence or absence of CIN of any grade or ICC (CIN+) using Pearson's χ^2^ test or Fisher's exact test when appropriate for qualitative variables and t-test or Kruskall-Wallis test for quantitative variables. A logistic regression model was used for univariate and multivariate analyses of the factors associated with CIN+. For the multivariate analysis, a stepwise descending procedure was applied to derive the model that best predicted the presence of any CIN+. The goodness of fit of the model was assessed using the Akaike Information Criterion (AIC), a lower value of the AIC suggesting a better prediction of the model. All relevant potential confounders were included in the initial multivariate model. Confounders that were not significantly associated with the presence of CIN+ and did not add any significant prediction to the model based on the AIC were sequentially removed. Although, no significant interaction was reported between ART use, the most recent CD4 count and the presence of CIN+, the model was secondarily stratified according to ART use to better explore the respective role of immunological status and ART on the presence of CIN+. Proportions and *Odds Ratio* (OR) estimates were reported with their 95% Confidence Interval (95% CI). All statistical analyses were performed using SAS software 9.2 (SAS Institute Inc. NC. USA).

## Results

Of the 3,090 HIV-positive women who attended the cervical screening consultations, 92 (3.0%) were not eligible. The median age of the 2,998 remaining women was 36 years [interquartile range (IQR): 32–42] and 2,267 (75.6%) were on ART. The median duration of follow-up in HIV care was three years [IQR: 1–5]. Among ART users, the median duration on ART was 2 years [IQR: 1–4]. The overall median most recent CD4 count was 452 [IQR: 301–621] cells/mm^3^, 439 [IQR: 282–616] cells/mm^3^ among ART users and 491 [IQR: 361–640] cells/mm^3^ among the untreated women (p<10^−4^). A total of 268 (9.0%) women were positively screened with suspected cervical lesions at visual inspection and referred for medical examination. Of these, 132 CIN+ (4.4%; 95% CI: 3.7–5.1) were identified. Biopsy results were as follows; 115 CIN of grade 1, five CIN of grade 2, nine CIN of grade 3 and three ICC. Table 1 presents the main characteristics of the women enrolled according to the presence of any CIN+. Table 2 summarizes the univariate and multivariate analysis of factors associated with the presence of CIN+.

**Table 1:** Main characteristics of HIV-infected women screened for cervical malignancies according to the presence or absence of cervical intraepithelial neoplasia of any grade (CIN+) in Abidjan, Côte d'Ivoire.

	No CIN+	Presence of CIN+	P	Total
	(n = 2,866)	(n = 132)		(n = 2,998)
**HIV clinic**, n (%)			0.58	
MTCT +	591 (20.6)	32 (24.3)		623 (20.8)
Cepref	1,303 (45.5)	56 (42.4)		1,359 (45.3)
CNTS	972 (33.9)	44 (33.3)		1,016 (33.9)
**Age in years**, median (IQR)	36 [32–42]	37 [31–41]	0.40	36 [32–42]
**Formal education**, n (%)			0.67	
No	725 (25.3)	32 (24.3)		757 (25.3)
Primary school	926 (32.3)	39 (29.5)		965 (32.2)
Secondary and over	1,213 (42.4)	61 (46.2)		1,274 (42.5)
**Tobacco use** *	43 (1.5)	1 (0.8)	0.49	44 (1.5)
**Hormonal contraceptive use** (current)	45 (1.6)	0 (0.0)	0.26	45 (1.5)
**Marital status**			**0.02**	
Married, cohabitant	1,298 (45.3)	46 (34.9)		1,344 (44.8)
Single, divorced, widowed	1,568 (54.7)	86 (65.1)		1,654 (55.2)
**Age at first sexual intercourse**, n (%)			0.66	
≥16 years	1,949 (69.3)	93 (71.0)		1,946 (69.2)
<16 years	863 (30.7)	38 (29.0)		866 (30.8)
**Lifetime number of sexual partners**			0.21	
<5	1,247 (43.8)	65 (49.2)		1,312 (44.0)
≥5	1,602 (56.2)	67 (50.8)		1,669 (56.0)
**Parity**, n (%)			**0.04**	
Nulliparous	448 (15.6)	10 (7.6)		458 (15.3)
Primiparous	687 (24.0)	35 (26.5)		722 (24.1)
Multiparous	1731 (60.4)	87 (65.9)		1818 (60.6)
**ART exposure** (years)			**0.04**	
Never on ART	705 (24.9)	26 (19.7)		731 (24.6)
≥1	906 (32.0)	54 (40.9)		960 (32.4)
≥2–3	617 (21.8)	33 (25.0)		650 (21.9)
≥4	606 (21.3)	19 (14.4)		625 (21.1)
**If yes, current ART regimen**	2,161 (100.0)	106 (100.0)	0.44	2,267 (100.0)
D4T/3TC/NVP	573 (26.5)	23 (21.7)		596 (26.3)
D4T/3TC/EFV	125 (5.8)	7 (6.6)		132 (5.8)
AZT/3TC/NVP	397 (18.3)	23 (21.7)		420 (18.5)
AZT/3TC/EFV	315 (14.6)	13 (12.3)		328 (14.5)
Protease inhibitor based regimen	207 (9.6)	13 (12.3)		220 (9.7)
Other regimens	544 (25.2)	27 (25.4)		571 (25.2)
**HIV type**			0.68	
HIV-1	1,694 (59.1)	78 (59.1)		1,772 (59.1)
HIV-2 or dually reactive	79 (2.8)	2 (1.5)		81 (2.7)
Unknown	1,093 (38.1)	52 (39.4)		1,145 (38.2)
**Baseline clinical stage**			0.94	
CDC A & B and/or 1,2 WHO	1,513 (52.8)	69 (52.3)		1,582 (52.8)
CDC C and/or 3,4 WHO	962 (33.6)	46 (34.8)		1,008 (33.6)
Unknown	391 (13.6)	17 (12.9)		408 (13.6)
**Baseline CD4 count**, median (IQR)†	295 [160–464]	249 [107–378]	**<10^−2^**	291 [156–461]
**Most recent CD4 count**, median (IQR)‡	458 [307–629]	340 [216–500]	**<10^−4^**	452 [301–621]

*Current or former use of smoked and/or chewed tobacco.

† CD4 count (cells/mm3) measured during first clinical follow-up for HIV infection.

‡ Last known CD4 count (cells/mm3) measured prior or during the cervical cancer screening visit.

Abbreviations: CIN/ICC Cervical Intraepithelial Neoplasia/Invasive Cervical Cancer ART Antiretroviral Treatment.

**Table 2:** Determinants of cervical intraepithelial neoplasia of any grade (n = 132) in 2,998 HIV-infected women in Abidjan, Côte d'Ivoire. The IeDEA West Africa collaboration.

			Univariate analysis			Final multivariate analysis	
Variables	n/N	OR	95% CI	P	OR	95% CI	P
**Age category** (years)				0.10			0.08
≥25–34	46/1,211	1			1		
≥35–44	68/1,265	1.4	1.0–2.1		1.3	0.9–2.0	
≥45–54	13/434	0.8	0.4–1.5		0.6	0.3–1.2	
≥55–65	5/88	1.5	0.6–3.9		1.2	0.5–3.3	
**Marital status**				0.02			<10^−2^
Couple (married, cohabitant)	46/1,344	1			1		
Single (alone, divorced, widowed)	86/1,654	1.5	1.1–2.2		1.6	1.1–2.4	
Parity				0.01			<10^−2^
No	10/458	1			1		
Yes (≥1)	122/2,540	2.3	1.2–4.3		2.5	1.3–4.9	
**Antiretroviral exposure** (years)∫				0.05			0.26
Never	26/731	1			1		
≥1	54/992	1.6	1.0–2.6		1.0	0.6–1.7	
≥2–3	33/650	1.4	0.9–2.4		1.1	0.6–2.0	
≥4	19/625	0.8	0.5–1.5		0.6	0.3–1.2	
**Baseline CD4 count** (cells/mm^3^)*				0.02			0.50
≤200	56/965	1			1		
>200–350	37/768	0.8	0.5–1.2		1.0	0.6–1.6	
>350	36/1,181	0.5	0.3–0.8		0.7	0.5–1.2	
Unknown	3/84	0.6	0.2–1.9		0.5	0.1–4.0	
**Most recent CD4 count** (cells/mm^3^)†				<10^−4^			<10^−3^
≤200	31/346	1			1		
>200–350	36/621	0.6	0.4–0.9		0.6	0.4–1.0	
>350	62/1,957	0.3	0.2–0.5		0.3	0.2–0.6	
Unknown	3/74	0.4	0.1–1.4		0.8	0.1–5.8	

∫ Comparison of CD4 count (cells/mm^3^) according to ART duration (median [interquartile range]): ≥1 years: 358 [212–532], ≥2–3 years: 477 [313–650], ≥ 4 years 512 [366–691] (p<10^−4^).

*CD4 count (cells/mm3) measured during first clinical follow-up for HIV infection.

† Last known CD4 count (cells/mm3) measured prior or during the cervical cancer screening visit.

In univariate analysis, a high CD4 count at first HIV care visit was protective against the presence of CIN+: >350 cells/mm^3^ (OR: 0.5; 95% CI: 0.3–0.8) (Ref: CD4 <200 cells/mm^3^) (*p* = 0.02). An even stronger association was found between CIN+ and the most recent CD4 count measure: >350 cells/mm^3^ (OR: 0.3; 95% CI: 0.2–0.5) or [200–350] cells/mm^3^ (OR: 0.6; 95% CI: 0.4–0.9) (Ref: CD4 <200 cells/mm^3^) (*p*<10^−4^). An association of borderline significance (p = 0.05) was found between ART use and the presence of CIN+. Compared to those not taking ART, being treated for less than two years was associated with the risk of being diagnosed as CIN+ (OR: 1.6; 95% CI 1.0–2.6) but the association disappeared for those on ART between 2 and 4 years (OR: 1.4; 95% CI 0.9–2.4) and over 4 years (OR: 0.8; 95% CI 0.5–1.5) (*p* = 0.05).

In the final multivariate analysis, the presence of CIN+ was associated with a most recent CD4 count >350 cells/mm^3^ (OR: 0.3; 95% CI: 0.2–0.6) or ≥200–350 cells/mm^3^ (OR 0.6; 95% CI 0.4–1.0) (Ref: <200 cells/mm^3^ CD4), parity ≥1 (OR: 2.5; 95% CI: 1.3–4.9) and being single (OR: 1.6; 95% CI: 1.1–2.4) whereas ART use was no longer associated (*p* = 0.26).

In a multivariate analysis restricted to ART users (N = 2267, 75.6% of the sample), CIN+ (n = 106) was significantly associated with a most recent CD4 count >350 cells/mm^3^ (OR: 0.4; 95% CI: 0.2–0.7) or ≥200–350 cells/mm^3^ (OR: 0.5; 95% CI 0.3–1.0) (Ref: CD4 <200 cells/mm^3^) (p<10^−2^). Among HIV-infected women that never experienced ART, CIN+ was also significantly associated with a most recent CD4 count >350 cells/mm^3^ (OR: 0.1; 95% CI: 0.0–0.5) (Ref: CD4<200 cells/mm^3^) (p = 10^−2^).

Women infected with the HIV-2 type or dually reactive to HIV-1 and HIV-2 did not experience more CIN+ lesions than women infected with HIV-1 only in univariate analysis (p = 0.69) and this variable was thus not kept in the multivariate analysis.

## Discussion

In our study, immunodeficiency (most recent CD4 count <350 cells/mm^3^) was identified as the main determinant of the presence of CIN+ in HIV-infected women and this association remained although with a lower strength among those on ART. Previous studies have reported an association between the presence of cytological abnormalities and a severe immunodeficiency (most recent CD4 count measure <200 cells/mm^3^) among HIV-positive women in sub-Saharan Africa [11]–[13]. Nevertheless, these studies, conducted in a period of limited access to ART, were not based on a histological assessment of cervical premalignant lesions. Infection with carcinogenic HPVs has been linked with immunodeficiency in previous studies suggesting that a higher risk of HPV acquisition and/or persistence might lead to more frequent premalignant and malignant cervical lesions [12], [14]–[17]. In Rwanda, Anastos *et al* studied the determinants of high grade CIN in 454 women not treated with ART and dually infected with HIV and Human Papillomavirus (HPV). A CD4 count decrease was associated with CIN of grade 3 in a subset of 241 women infected with non-HPV16 carcinogenic HPV suggesting that even among women already harbouring carcinogenic HPVs, immunodeficiency was associated with the presence of high grade CIN [18]. A recent report from Kenya found a significant and inverse association between a most recent CD4 count over 500 cells/mm^3^ and the presence of high grade CIN (adjusted OR: 0.42, [95% CI 0.22–0.80]) among HIV-infected women, but no association was found among ART users [19]. We confirmed this association in a large population of HIV-infected women in care in West Africa. Furthermore, our report also highlighted that in a period of expanding access to ART, thus with less severely immune-compromised women at entry and more importantly during follow-up, immunological status remained a major determinant of CIN+, although ART use seemed to attenuate this association.

Baseline CD4 count at the time of first clinical follow-up which could be interpreted as a proxy for nadir CD4 count, was not found to be significantly associated with CIN+ in our multivariate model. This result contrasts with other reports from resource-constraint settings [19], [20]. In Brazil, an HIV care clinic reported that a nadir CD4 count <350 cells/mm^3^ was found to be the main factor associated with CIN2+ among 366 newly enrolled HIV-infected women (Ref: CD4 count nadir ≥350 cells/mm^3^) (adjusted Prevalence Ratio: 6.0; 95% CI: 1.5–24.3). These somewhat discordant findings raise questions on the respective role of cumulative time spent on immunodeficiency as well as the CD4 nadir reached before ART is initiated. As we do not have any further information on the respective timing of acquisition of HIV infection and of the occurrence of premalignant cervical lesions, we were not able to explore the precise influence of these two parameters on the occurrence of CIN+. Prospective cohort studies represent the only methodology to provide a final and direct assessment of the impact of immunodeficiency on the occurrence of cervical malignancies in the ART era.

Reports assessing the impact of ART on the occurrence of CIN+ in resources-limited settings have been conflicting. So far, reports from Nigeria and South Africa found no association between squamous intraepithelial lesions (SILs) of the cervix and ART exposure [11], [12]. In India, Saharsrabuddhe *et al* found that HIV-infected women currently receiving ART were at higher risk of presenting a CIN compared to untreated women [21]. In Kenya, DeVuyst *et al* found no significant association between ART use and the presence of high grade CIN [22]. Several hypotheses have been raised to foresee the potential role of ART on the occurrence of CIN. Through immunological recovery, ART could enhance oncogenic HPV clearance in HIV-positive women and thus regression of premalignant cervical lesions. Conversely, as women accessing to ART have a longer life expectancy [2], other authors have speculated that higher rates of cervical malignancy would be reported in HIV-infected women as ART continues to expand. The association found in our study between ART use and CIN+ in univariate analysis is not totally surprising. Indeed, as ART users presented with a significantly lower median most recent CD4 count measure compared to those not treated, they were expected to present with a higher rate of CIN+, especially those treated for a short duration. In ART users, the decrease of CIN+ observed with the increased duration on ART suggests that, through immune recovery, ART use might have a protective effect against HPV infection and premalignant cervical lesions. This association was secondarily shaded by the introduction of the most recent CD4 count variable in the multivariate model.

However, the cross-sectional nature of the study and the relatively long natural history of cervical malignancies are currently limiting our ability to accurately assess the impact of ART on the development of cervical malignancies. A report from Minkoff *et al* showed that taking into account its effectiveness and adherence, the use of ART was significantly associated in a reduction of incident squamous intraepithelial lesions of the cervix in a prospective cohort of 286 HIV-infected women in the United-States of America [23].

Contrary to previous reports which showing a higher risk of CIN+ among women who smoke, the proportion of smokers according to the presence of CIN+ was not statistically different here [24]. As the impact of tobacco on HPV infection and cervical malignancies might be essentially mediated through an impaired immune response, the role of tobacco among HIV-positive women might have been shaded.

We found that single or divorced women were more likely to harbour CIN+ compared to married women. As neither the reported number of lifetime sexual partners nor the age at first sexual intercourse was found to be associated with the presence of CIN+, we believe that the marital status could be interpreted as a proxy for the current number of sexual partners and less subject to prevarication and recall biases. Parity was also associated with the presence of CIN+ in multivariate analysis. This association was expected as parity has been reported as a risk factor for ICC in populations of unknown HIV status [25], as well as among HIV-positive women [18].

Before the advent of ART in sub-Saharan Africa, HIV-2 infection had been identified as a potential risk factor for the occurrence of premalignant and malignant cervical lesions [26], [27]. These authors had originally proposed that this association could be related to a better survival related to the longer natural history of HIV-2 infection compared to HIV-1, thus enabling the occurrence of CIN+. We found ourselves no association between HIV types and CIN. The expanding access to ART might have shaded the relative impact of HIV type alone initially described prior to ART scale-up in West Africa.

### Limitations

The main outcome variable of this analysis was CIN of any grade, including low-grade lesions because of the limited number of high grade cervical lesions (CIN 2/3 or ICC). As low grade lesions (CIN 1) have the ability to spontaneously regress, this outcome might not reflect the true population at risk of developing ICC. Considering the HIV subtypes, there were limited numbers of HIV-2 infections limiting therefore our ability to detect any association with the presence of CIN+. Using visual inspection methods to assess the presence of precancerous lesions exposes to misclassification due to its limited sensitivity and specificity compared to colposcopy with directed biopsy. As there are currently no affordable alternative screening method and as conventional cytology in field conditions has not demonstrated its superiority compared to visual inspection, we support this public health approach as the most appropriate one for targeting HIV-infected women in care and believe this has not interfered with our research findings.

## Conclusion

In a context of expanding roll-out of ART in West Africa, immunodeficiency remains a major determinant of the occurrence of CIN+ among HIV-infected women although the frequency of CIN+ lesions decreased with the duration on ART. In resource-limited settings where cervical cancer screening programs are challenging to organize, immunological preservation and reconstitution might partly prevent the occurrence of premalignant cervical lesions. However, cervical cancer prevention, including screening of premalignant lesions as well as HPV vaccination, remains of utmost priority especially in HIV-infected women in a context of expanding roll out of ART in West Africa.
